# Johnny Cakes

**Published:** 1854-12

**Authors:** 


Johnny Cakes.—Scald a quart of sifted Indian meal with sufficient water to make a thick batter; stir in a tablespoonful of salt; flour the hands well, and mould it into small cakes; fry them in fat enough nearly to cover them. When brown upon the under side they should be turned. It takes about twenty minutes to cook them. When done, split and butter them.

